# Cobll1: A new player in CML

**DOI:** 10.18632/oncotarget.21705

**Published:** 2017-10-10

**Authors:** Hongtae Kim, Dong-Wook Kim, Kyungjae Myung

**Affiliations:** Kyungjae Myung: Center for Genomic Integrity Institute for Basic Science, Ulsan National Institute of Science and Technology, Ulsan, Republic of Korea; Dong-Wook Kim: Leukemia Research Institute, The Catholic University of Korea, Seoul, Republic of Korea; Hongtae Kim: Department of Biological Science, Sungkyunkwan University, Suwon, Republic of Korea

**Keywords:** Cobll1, CML, TKI, BP, HSC

**Figure 1:**
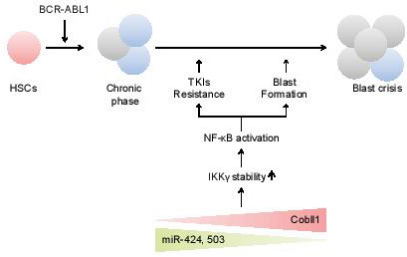
Model of drug resistance and progression in CML

Chronic myeloid leukemia (CML) is a malignant cancer derived from hematopoietic stem cells. CML is induced by an oncogenic BCR-ABL1 fusion protein, which has constitutive tyrosine kinase activity. The BCR-ABL1 fusion protein is produced from the fused Philadelphia chromosome formed by the translocation of chromosomes 9 and 22 [1]. Multiple pharmaceutical companies have developed specific inhibitors of the BCR-ABL1 tyrosine kinase inhibitor (TKI). Imatinib, the first generation of such TKIs, has been used to treat CML [2]. Although imatinib could treat many patients with CML, some CML cells have developed resistance to imatinib. Resistance to imatinib allows CML to progress from the initial chronic phase (CP) to the late advanced blast phase (BP) [3]. To treat such resistant CML, a second-generation TKI, nilotinib, was developed. However, CML has developed resistance to nilotinib as well. Multiple studies have revealed that resistance to TKIs, including nilotinib, could be BCR-ABL-dependent [4] and -independent [5]. BCR-ABL-dependent resistance typically results from a point mutation or amplification of BCR-ABL. However, the mechanism by which BCR-ABL-independent resistance to TKI develops is still not clearly understood. In a recent study published in *Leukemia,* we found that the expression of Cobll1 was strongly correlated to drug resistance and blastic transformation in CML [6]. We revealed a novel mechanism involving Cobll1 for TKI resistance and progression of CML to BP. Cobll1 was found to be selectively overexpressed in BC cells from the bone marrow and peripheral blood in paired or relapsed CML patients. The expression of Cobll1 is dependent on the progression of CML to the BP. The importance of Cobll1 in TKI resistance was proved by the significant increase in TKI resistance in CML cells in the CP with ectopic expression of Cobll1 and the re-sensitization of CML cells in the BP by Cobll1 knockdown. Consistently, the survival rate of CML patients in the BP with high Cobll1 expression was lower than that of CML patients in the BP with low Cobll1 expression. These results indicated that Cobll1 is an important factor for determining the survival of CML patients in the BP. Furthermore, we demonstrated that Cobll1 was highly expressed in CD34+/CD38- or CD34+/CD38+ primitive stem cell populations from CML patients in the BP. Consistently, we found that the zebrafish paralog, Cobll1b, was important for normal hematopoiesis during embryonic development. The high levels of Cobll1 during CML progression appears to be achieved by an increase in Cobll1 mRNA, which is strongly correlated with the downregulation of miR-424 and miR-503 during CML progression. Consistently, when miR-424 or miR-503 was overexpressed in K562 cells, Cobll1 was significantly reduced, and the cells become sensitive to nilotinib. Increased Cobll1 in CML stabilizes IKKg to activate the NF-kB signaling pathway, leading to nilotinib resistance and progression to BP. The model for Cobll1 function is as follows (Figure 1). High levels of Cobll1 expression promotes the transformation of CML and generates drug resistance by increasing IKKγ stability, which results in the activation of the canonical NF-κB pathway. The importance of Cobll1 in CML progression and TKI resistance revealed in our recent studies suggests a novel Cobll1-targeting strategy for treating TKI-resistant CML. However, some unanswered questions need to be addressed before such a novel strategy can be developed: (i) Can there be a small molecule targeting Cobll1 function? (ii) Half the BC cells in the study were Cobll1-negative. What are the pathway(s) that regulate BC progression and TKI resistance in these cells? (iii) How is miR-424, which controls the expression of Cobll1, increased in BC?
